# Cardiac 18F-FDG Positron Emission Tomography: An Accurate Tool to Monitor *In vivo* Metabolic and Functional Alterations in Murine Myocardial Infarction

**DOI:** 10.3389/fcvm.2021.656742

**Published:** 2021-05-25

**Authors:** Maximilian Fischer, Mathias J. Zacherl, Ludwig Weckbach, Lisa Paintmayer, Tobias Weinberger, Konstantin Stark, Steffen Massberg, Peter Bartenstein, Sebastian Lehner, Christian Schulz, Andrei Todica

**Affiliations:** ^1^Medizinische Klinik und Poliklinik I, Klinikum der Universität München, Ludwig-Maximilians-Universität, Munich, Germany; ^2^DZHK (German Centre for Cardiovascular Research), Partner Site Munich Heart Alliance, Munich, Germany; ^3^Department of Nuclear Medicine, University Hospital, Ludwig-Maximilians-Universität (LMU) Munich, Munich, Germany; ^4^Ambulatory Healthcare Center Dr. Neumaier & Colleagues, Radiology, Nuclear Medicine, Radiation Therapy, Regensburg, Germany

**Keywords:** cardiac positron emission tomography, 18F-FDG, myocardial infarct, cardiac remodeling, heart function

## Abstract

Cardiac monitoring after murine myocardial infarction, using serial non-invasive cardiac 18F-FDG positron emissions tomography (PET) represents a suitable and accurate tool for *in vivo* studies. Cardiac PET imaging enables tracking metabolic alterations, heart function parameters and provides correlations of the infarct size to histology. ECG-gated 18F-FDG PET scans using a dedicated small-animal PET scanner were performed in mice at baseline, 3, 14, and 30 days after myocardial infarct (MI) by permanent ligation of the left anterior descending (LAD) artery. The percentage of the injected dose per gram (%ID/g) in the heart, left ventricular metabolic volume (LVMV), myocardial defect, and left ventricular function parameters: end-diastolic volume (EDV), end-systolic volume (ESV), stroke volume (SV), and the ejection fraction (EF%) were estimated. PET assessment of the defect positively correlates with post-infarct histology at 3 and 30 days. Infarcted murine hearts show an immediate decrease in LVMV and an increase in %ID/g early after infarction, diminishing in the remodeling process. This study of serial cardiac PET scans provides insight for murine myocardial infarction models by novel infarct surrogate parameters. It depicts that serial PET imaging is a valid, accurate, and multimodal non-invasive assessment.

## Introduction

Coronary artery disease (CAD) and myocardial infarction (MI) are leading causes of morbidity and mortality in western society (1, 2). In the last decades, eminent progress in disease prevention, diagnostics, and therapy of cardiovascular diseases have emerged. Mainly, innovative imaging applications, e.g., magnetic resonance imaging (MRI) or positron emissions tomography (PET), help optimize the diagnostics and treat cardiovascular patients by providing accurate and specific imaging modalities of the heart. This progress contributes to the rising survival of cardiovascular patients after myocardial infarct (3). Nevertheless, the count of sequel burdens such as ischemic heart disease, heart failure, and cardiac dysrhythmia is rising (4). A central issue after myocardial infarct is the irreversible loss of cardiomyocytes. Thus, leading to ischemic heart failure with reduced cardiac function and increasing left ventricular volumes. As a consequence of the acute injury, the heart adapts to cardiomyocytes' loss by remodeling, which is the sum of ventricular dilation, cardiomyocyte hypertrophy, and fibrotic scar formation (5, 6). The process of remodeling divides into an acute and a chronic phase (7). With the rise in experimental approaches such as antibody-mediated modulation of inflammation in atherosclerosis, there is a high demand for innovative methods and parameters to monitor new therapies' efficacy and impact (8, 9). The innovative small animal PET imaging is an imaging modality in different disease models, e.g., ischemic and dilated cardiomyopathy (10, 11), pressure-overload left ventricular hypertrophy (12) and arrhythmogenic right ventricular cardiomyopathy (ARCV) (13). The evaluation of cardiac function and parameters of the heart muscle can accurately be monitored by serial non-invasive cardiac PET imaging (14, 15).

Several radionuclide tracers were already evaluated and provide insight in the assessment of murine myocardial infarction. Radionuclide tracers in rat myocardial infarcts such as cardiac 18F BMS747158-02 PET proved to estimate the PET defect size and left ventricular systolic and diastolic volume, that could provide a promising technique for the evaluation of MI in clinical trials (16). Using Rubidium-82 PET imaging in rats enables fast and time-efficient myocardial perfusion imaging after MI (17). Sequential evaluation of 18F-FDG PET and MRI in rat myocardial infarction previously showed to be a suitable approach to monitor cardiac remodeling in a multimodal way (18). However, novel intracellular target proteins or RNA are mainly assessed in transgenic and knockout mice models and combination of several imaging modalities are cost and time consuming. To further overcome the several combinations of PET, MRI, and computer tomography (CT) for small-animal imaging, this study aimed to simultaneous determine robust metabolic and as well functional cardiac parameters in serial mouse 18F-FDG PET imaging after MI to monitor tissue remodeling.

## Materials and Methods

### Animals

Male C57/BL6 mice were purchased from Charles River Laboratories (Sulzfeld, Germany). According to the Guideline for the Care and Use of Laboratory Animals published by the U.S. National Institutes of Health (NIH publication no. 85-23, revised 1996), animal care and all experimental procedures were performed. Acute myocardial injury was induced in 10 weeks old (group 1) and 18 weeks-old male (group 2) (see Supplementary Table 1). Two mice died after MI in group 1; one mouse did in group 2 after MI induction. C57BL/6 mice by total surgical ligation of the left anterior descending artery, as described previously (19). The experimental groups are listed in supplementals. All animals received humane care. Study protocols complied with the institution's guidelines and were approved by the Government's animal ethics committee (Gz. 55.2-1-54-2532-45-13).

### *In vivo* Cardiac PET Imaging

ECG-gated 18F FDG-PET scans were performed at baseline, post-operation day 3, 14, and 30 using a dedicated small-animal PET scanner (Inveon Dedicated PET, Preclinical Solutions, Siemens Healthcare Molecular Imaging, Knoxville, TN, USA) as described previously (12, 20). The animals had free access to food and water until before the scan, as described previously (10, 12, 20, 21). Anesthesia was induced (2.5%) and maintained (2%) with isoflurane delivered in pure oxygen at a rate of 1.5 L/min *via* a face mask. The core body temperature was maintained within the normal range using a heating pad and monitored by a rectal thermometer. Neonatal ECG electrodes (3M, St. Paul, MN, USA) were placed on both forepaws and the left hind paw. After placing an intravenous catheter into a tail vein, ~20 MBq of 18F-FDG was injected in a volume of ~0.1 ml. The catheter was then flushed with 0.05 ml of saline solution. Animals remained anesthetized during the entire scan and were placed in a prone position within the PET scanner. A three-dimensional PET recording in list mode was initiated 30 min after injection of the tracer and lasted for 15 min. For attenuation and scatter correction, a 7-min transmission scan was performed with a rotating [57Co] source immediately after each PET scan, as described previously (10). Recovery from anesthesia and the PET scan was monitored closely in the home cage with a veterinarian monitoring. The recorded data was then processed with the Inveon Acquisition Workplace (Siemens Medical Solutions, Knoxville, TN, USA). 18F-FDG list-mode acquisitions were reconstructed, as described previously (10). Reconstruction was performed using an OSEM 3D algorithm with 4 iterations (16 subsets) and a MAP 3D algorithm with 32 iterations (16 subsets) in a 128 ×128 matrix with a zoom of 211%. Data was either reconstructed as a static image or as a cardiac gated image with 16 bins, normalized, corrected for randoms, dead time, and decay as well as attenuation and scatter.

### PET Image Analysis

Analysis of PET images was performed by the Inveon Research Workplace (Siemens Medical Solutions) described previously (22, 23).

Inveon Research Workplace was used for assessing the cardiac %ID/g and LVMV from static images. A cubic volume of interest (VOI) was drawn around the left ventricle, and a threshold value excluding the 30% least hot voxels was applied. Correct VOI placement was verified in three projections (axial, sagittal, and coronal) (12). Our previous publication (24) shows that LVMV depends on mice age, and there are significant differences between 10 and 18 weeks old mice. We could not detect this aspect in the evaluation of %ID/g. The male age-matched control mice partially derive from a healthy murine database that was acquired during the same period (24).

ECG trigger signal accuracy was retrospectively verified using in-house software programmed in MATLAB (The Mathworks, Natick, USA) (25).

The parameters of PET infarct size (or defect) were calculated from static images with QPS® (Cedars-Sinai, Los Angeles, CA, USA) using a normative database, as described previously (26).

Infarct sizes were estimated with commercially available software (QPS® 2012) from static FDG PET scans by creating polar maps of the left ventricle. In this approach, the FDG uptake in a given region is quantified as the percentage of the maximum tracer uptake in the polar map. The individual polar map is then compared to the average polar map from our own normative database consisting of gender, strain, and age-matched animals. The extent of the abnormally viable myocardium is calculated as a percentage of the left ventricle surface area. Furthermore, the severity of the abnormal viability is calculated in units of standard deviations below the normal tracer uptake. Extent and severity are then combined into a single parameter corresponding to the infarct size expressed as percentage of the left ventricular surface area. Left ventricular function parameters: EDV, ESV, SV, and EF(%), were calculated from ECG-gated images using QGS® (Cedars-Sinai, Los Angeles, CA, USA), as described previously (10, 22).

### Histology

Mice were sacrificed on days 3 and 30 after permanent LAD ligation, and the hearts were excised. After fixation in 4% phosphate-buffered formalin, hearts were cut into 2 mm thick slices and embedded in paraffin. Four micrometer thick sections were cut and mounted on positively charged glass slides. Standard histological procedures (hematoxylin/eosin and Masson's trichrome) were performed. Infarct size was determined as described previously (20).

### Statistical Analysis

All results were expressed as means with standard deviation. ANOVA analysis with Sidak's multiple comparisons and paired Student's *t*-test were used as indicated. For groups without normal distribution, the Wilcoxon signed-rank or the Mann–Whitney *U*-test was applied. The differences were considered statistically significant at a *P*-value of 0.05.

## Results

### Positive Correlation of PET and Histology Infarct Area in Hearts After Myocardial Infarction

To investigate the relationship between PET scan and histology assessment after MI, we analyzed the established animal model of permanent LAD ligation at post-operation day 3 and day 30. In MI, an experimental cardiac injury model of the left ventricle, the immediate loss of viable tissue triggers cardiac remodeling.

**Figure 1:**
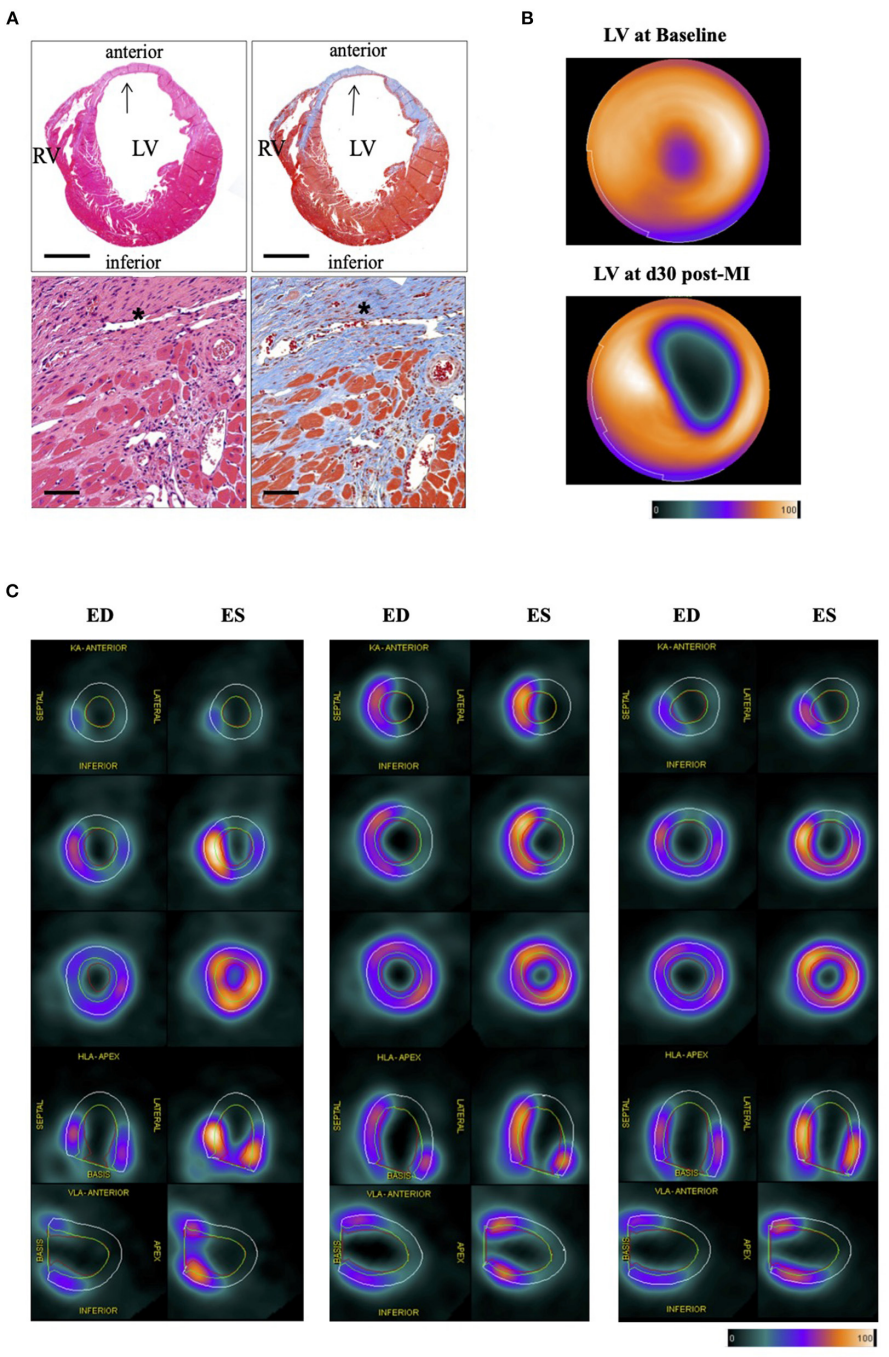
Histological and PET imaging of murine myocardial infarction. **(A)** Histology section of the heart 30 days after LAD ligation. HE-Staining (left) and Masson's trichrome staining (right). Stars and arrows indicate the magnified infarct scare area in the left ventricular (LV) wall (blue). Right ventricle (RV). Bar equals 100 and 50 μm, respectively. **(B)** Representative bulls-eye image of the left ventricle of healthy (upper image) and infarcted left ventricle (lower image) showing the defect of the infarct. Color Scale from QPS: Cool and in percentage range. **(C)** Panel shows PET images of three representative infarct hearts (left, middle, and right panel) each in end-diastole (ED) and end-systole (ES) in different axes at various levels of the left ventricle. ED volume (green line) and ES volume (red line) depicting the motion deficit in the infarct area (KA, coronary axis; VLA, vertical long axis; HLA, horizontal long axis). EF% from left to right: 40%, 34%, 42% (QGS); Defect% (QPS) from left to right: 48%, 44%, 40%. Color Scale from QGS: Cool as used in **(B)**. For the segmental defect and motion deficit of the three representative mice, please see Supplementary Figures 2A,B, respectively.

For histological infarct assessment, heart sections were stained and evaluated by microscopy at day 3 and day 30 post-operation (Figure 1A). Using the small animal PET scanner, we investigated the infarct area by the diminished uptake of 18F-FDG in the left ventricle in a three-dimensional evaluation (Figures 1B,C). Figure 1C is depicting three representative PET images in different axes for infarct hearts in end-diastole (ED) and end-systole (ES) representing the motion deficit of the infarct. The segmental defect scoring and corresponding segmental motion severity scoring further depicts the motion defect evaluated by QPS and QGS software (Supplementary Figures 2A,B).

**Figure 2:**
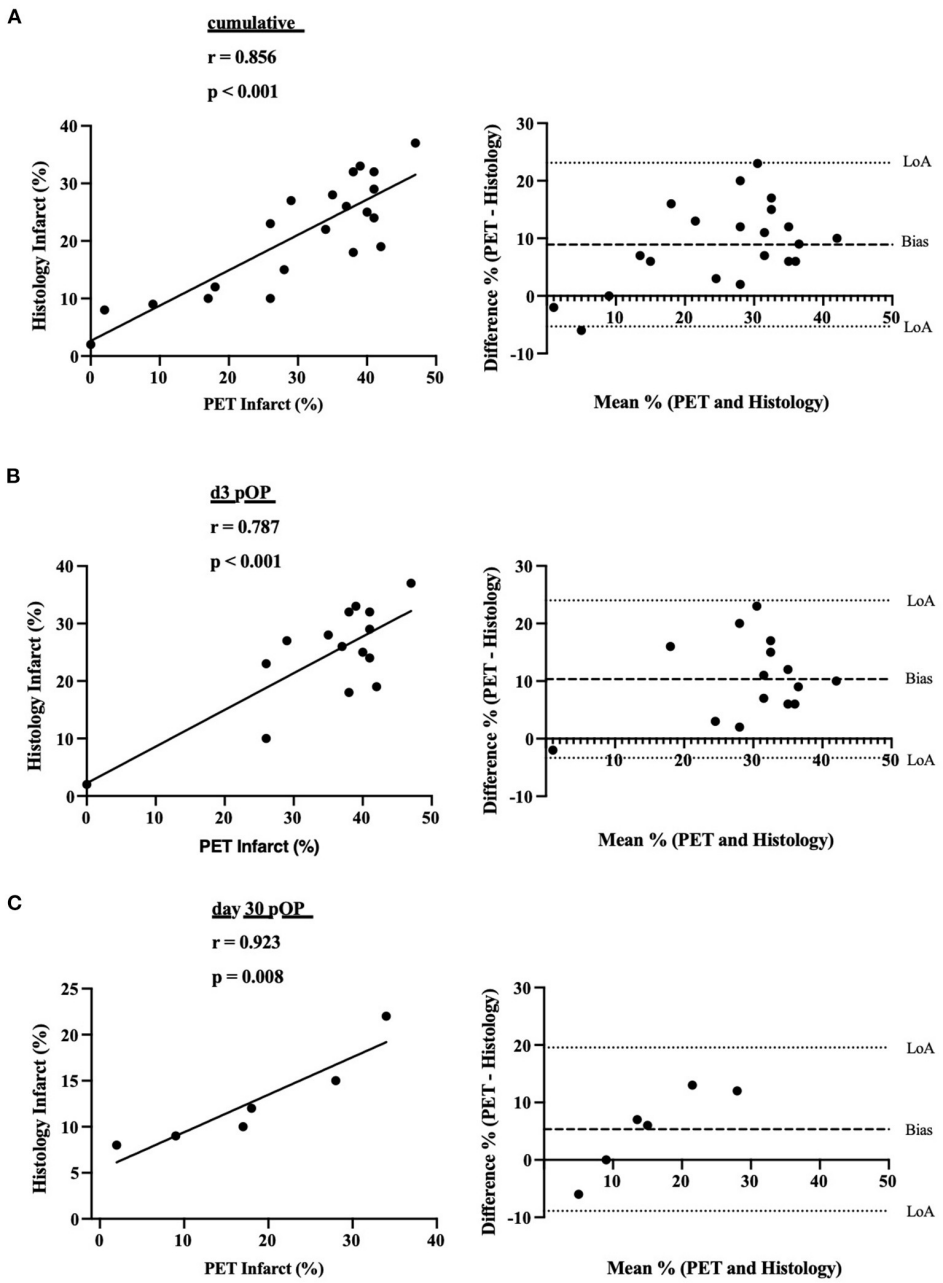
Scatter plots of the correlation between histological and PET infarct areas. **(A)** Cumulative correlations between histological and PET infarct area (in %). Corresponding Bland-Altman-plot on the right side. Dotted lines display the Limits of agreement (LoA) and correlation line. Dashed lines indicate bias. **(B)** Correlations between histological and PET infarct area (in %) at day 3 post-MI. Corresponding Bland-Altman-plot on the right side. **(C)** Correlations between histological and PET infarct area (in %) at day 30 post-MI. Corresponding Bland-Altman-plot on the right side.

Cardiac PET scans of experimental subgroups were performed at baseline, day 3, day 14, and day 30 post-operation to estimate metabolic and functional alteration in the remodeling process in mice of 10 and 18 weeks of age. Of note, mice were either scanned at baseline, day 3, or at baseline, day 3 and day 30 or baseline, day 14, and day 30. In total 15 mice (10 weeks old mice: *n* = 8, and 18 weeks old mice: *n* = 7) were used for correlation of PET infarct day 3 and histology. Six mice were evaluated for the correlation at day 30. (see Supplementary Table 1 and Supplementary Figure 1A for the experimental setting of mice at a different age). All mice were used for the baseline PET assessment, while subgroups mice were sacrificed for histology on day 3 and 30. Remarkably, the histological and PET infarct area's cumulative and subgroup correlations regarding the statistical correlation are highly significant. The cumulative correlation includes the evaluation at day 3 and day 30 post-infarct of mice, along with the corresponding Bland-Altman plots (*r* = 0.856, *p* < 0.001, Figure 2A). There is also a strong correlation between the two methods at early day 3 post-operation (*r* = 0.787, *p* < 0.001, Figure 2B). Similar results were obtained at day 30 (*r* = 0.923, *p* = 0.008, Figure 2C). Of note, only the 10 weeks old mice were assessed for histology on days 3 and day 30. These data suggest that PET measurements for assessing the cardiac infarct area are a valid tool for early damage and later evaluation of the remodeling scar at day 30 post-infarct.

### Early Loss of LVMV and Increase in Cardiac %ID/g After Myocardial Injury

**Figure 3:**
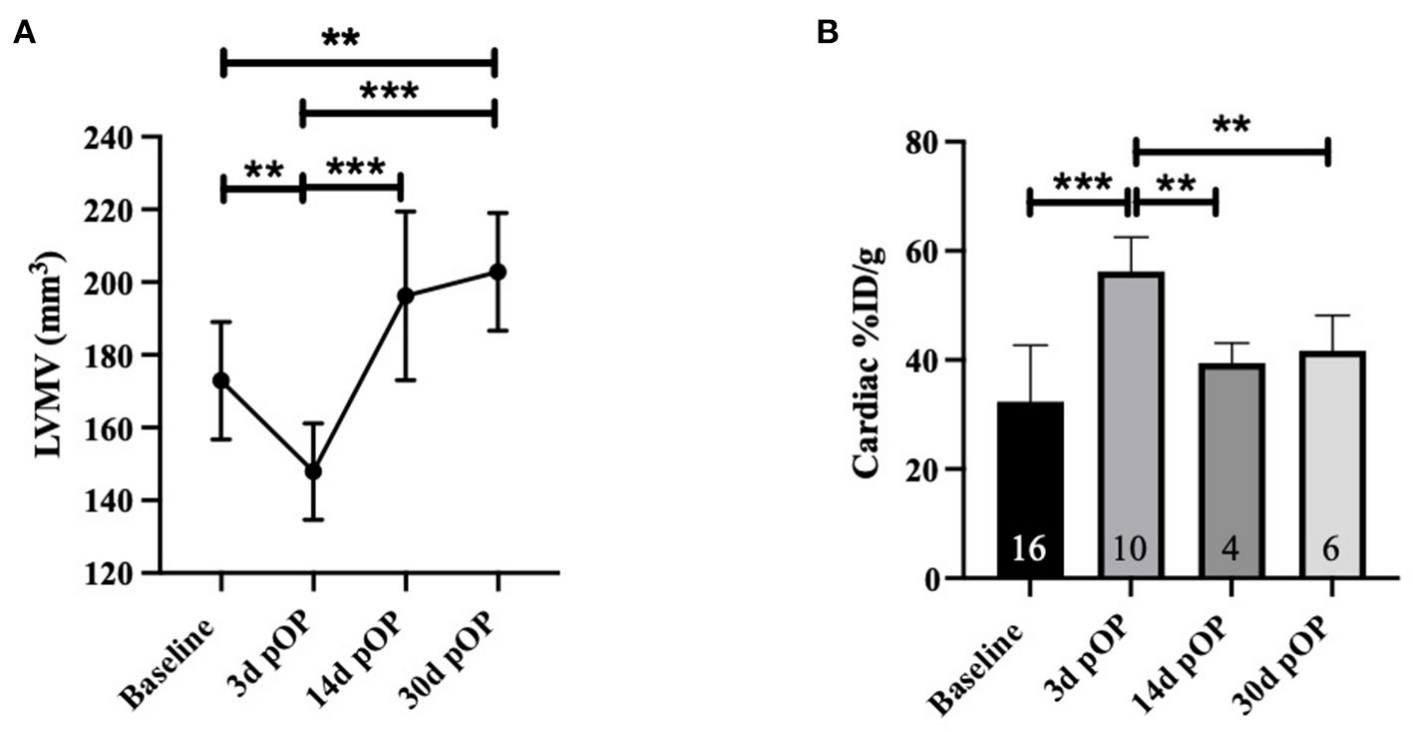
Alterations in LVMV and cardiac %ID/g in myocardial infarction in 10 weeks old mice **(A)** displays the LVMV and **(B)** for cardiac %ID/g at baseline, 3, 14- and 30-days post-MI. All groups: *n* = 4-16. All data represent mean ± SD. * *p* = 0.05, ** *p* < 0.01, *** *p* < 0.001.

Next, we assessed whether metabolic alterations in 18F-FDG uptake in the infarcted hearts could be observed. The LVMV and the cardiac %ID/g were estimated in the 10 weeks old mice at baseline and post-infarct days 3, 14, and 30. As to be expected, a significant drop in LVMV could be observed at 3 days post-operation (baseline MI vs. MI d3, *p* = 0.003, Figure 3A). This decrease in LVMV is regressive at 14 days (MI d3 vs. MI d14, *p* < 0.001). Additionally, compared to the baseline and 3 days post-infarct, the LVMV already significantly increased after 30 days (baseline MI vs. MI d30, *p* = 0.003; MI d3 vs. MI d30, *p* < 0.001) (see Table 1). Of note, despite higher LVMV at baseline at 18 weeks old mice, we could detect a similar drop in LVMV at day 3 post-MI (18 weeks vs. 18 weeks 3d pOP, *p* > 0.001, Supplementary Figure 1C). Regarding the cardiac %ID/g in the 10 weeks old mice, a parameter that resembles the ratio between the activity of the tracer detected in the cardiac tissue, and the total tracer activity injected, we also observed a significant increase in the early cardiac injury at day 3 (baseline MI vs. MI d3, *p* < 0.001, Figure 3B), whereas this increased cardiac uptake of 18F-FDG normalizes at day 14 (MI d3 vs. MI d14, *p* =0.009; MI d3 vs. MI d30, *p* =0.01). Again, similar results (18 weeks vs. 18 weeks 3d pOP, *p* < 0.001) for the 18 weeks old cohort are shown in Supplementary Figure 1C).

**Table 1:** PET measurements in 10 weeks old mice after myocardial infarction.

	**PET infarct/defect (%)**	**LVMV (mm^**3**^)**	**%ID/g**	**EDV (μl)**	**ESV (μl)**	**SV (μl)**	**EF (%)**
Baseline	2.2, 2.7	172.9, 16.2	32.1, 9.1	39.7, 8.8	6.9, 4.7	32.8, 5.2	83.9, 7.9
3d pOP	35.8, 8.8	147.9, 13.3	56.2, 6.3	46.0, 8.4	22.3, 6.4	23.9, 5.4	51.8, 8.6
14d pOP	24.5, 18.5	196.3, 23.2	39.4, 3.7	59.0, 10.9	30.0, 12.1	29.0, 9.9	49.8, 16.5
30d pOP	24.4, 9.5	202.8, 16.2	41.7, 6.5	60.2, 13.2	29.3, 14.3	31.0, 7.1	53.2, 14.6

*Values indicate Mean ± SD. Defect, LVMV, left ventricular metabolic volume; %ID/g, percentage of the injected dose per gram; EDV, end-diastolic volume; ESV, end-systolic volume; SV, stroke volume; EF, ejection fraction*.

### Reduced Ejection Fraction and Left Ventricular Dilatation After 30 Days Post-MI

**Figure 4:**
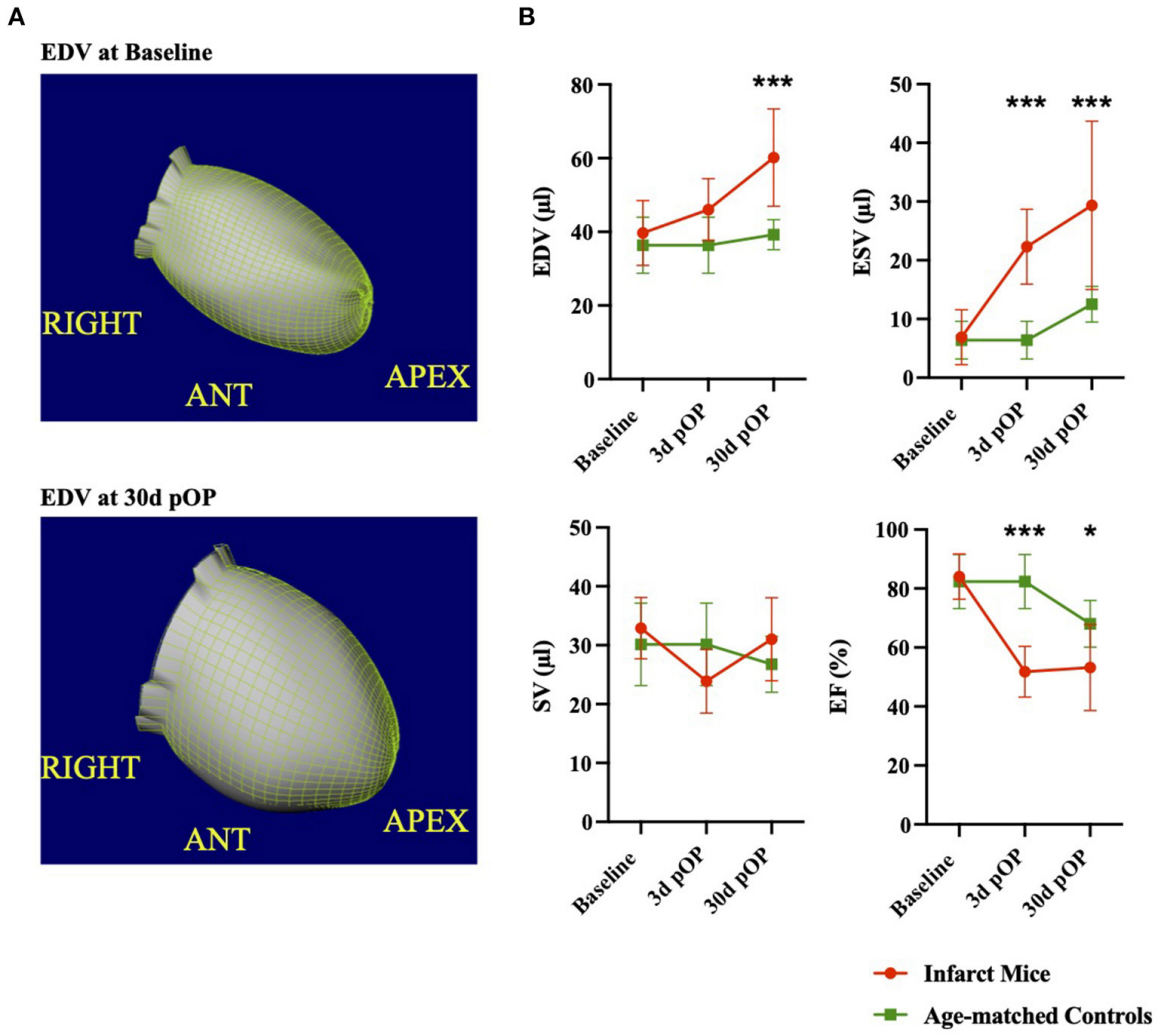
Evaluation of cardiac function after myocardial injury in comparison to age-matched littermates **(A)** illustrates three-dimensional reconstruction of the left ventricular EDV in the ROA view. Upper picture: EDV at baseline and lower picture at 30 days post-LAD ligation. **(B)** Comparison of cardiac PET parameters at baseline, 3, and 30 days post-MI. Data of healthy age-matched littermates are illustrated in green, and infarct mice are in red. All groups: *n* = 6-16. Data represent mean ± SD. * *p* < 0.05, ** *p* < 0.01, *** *p* < 0.001.

To determine the impact of MI on cardiac function (e.g., EDV, ESV, SV, and EF) *in vivo*, the ECG-gated 3D-PET scans were used at 3 and 30 days post-infarct and compared with healthy age-matched littermates to reduce the bias of age-related heart growth and hypertrophy. Figure 4A shows representative three-dimensional EDV at baseline and day 30 post-infarction. Regarding the EDV in healthy hearts, no difference could be detected from baseline to 30 days in the age-matched controls (baseline Control vs. Control d30, *p* = 0.875, Figure 4B left upper). In contrast, the infarcted hearts showed significantly higher EDVs than baseline and the age-matched healthy hearts at 30 days post-operation (Baseline MI vs. MI d30, *p* < 0.001; Control d30 vs. MI d30, *p* < 0.001). Similar results were obtained for the ESV in the infarcted hearts during the time (Baseline MI vs. MI d30, *p* < 0.001) and toward the age-matched controls (Control d30 vs. MI d30, *p* < 0.001). In contrast, the healthy age-matched mice did not differ from baseline (Baseline control vs. control d30, *p* = 0.131, Figure 4B right upper panel). We could not detect a change in SV at day 3 or 30 post-infarct (Baseline MI vs. MI d3, *p* = 0.093; baseline MI vs. MI d30, *p* = 0.472, (Figure 4B left lower panel). Overall, the SV did not change in either the infarcted or healthy age-matched mice regarding the baseline (Baseline control vs. control d30, *p* = 0.49). Notable, a significant drop in EF could be detected at 3 days between the infarcted and the age-matched controls (MI d3 vs. control d3, *p* < 0.001, Figure 4B right lower panel), that decrease remained at 30 days post-operation (MI d30 vs. control d30, *p* = 0.013). The cardiac function parameters were also recorded in the 18 weeks old experimental group at baseline and day 3. Similar results in EDV, ESV, SV, and EF were obtained (see Supplementary Figure 1C and Supplementary Table 2). In summary, these data demonstrate the pathological changes in cardiac function parameters over time compared to healthy age-matched littermates.

## Discussion

This study presents ample evidence that using small animal PET imaging is valid and appropriate to monitor cardiac infarcts at early and later stages by providing a good correlation for histological infarct area and assessing clinically significant cardiac function parameters.

Histological assessment is still a gold standard for infarct size evaluation, despite several disadvantages (27). However, histological approaches are not useful for the same animal's serial experiments due to the need to scarify the animals.

Therefore, accurate serial measurement of myocardial infarct areas and evaluation of cardiac function is on demand. Small animal PET imaging is one of the latest innovative methods. The advantage of PET infarct assessment is the three-dimensional approach that offers quantification of ventricular volumes and accurate non-invasive cardiac evaluation.

The *in vivo* PET assessment of myocardial infarction ligation is still a challenge in preclinical imaging and the search for valid tracers is ongoing. Previously, the usage of 99mTc-sestamibi and 13N-ammonia PET showed a valid assessment of cardiac function in healthy and infarct mice compared to MRI and contrast CT (28). Furthermore, Croteau et al. depicted a robust test and retest repeatability for mouse infarct assessment by using 11C-acetate micro-PET imaging (29).

While manually or semi-automatic drawing of the endocardial contours is time-consuming, an automated software-based evaluation by QPS/QGS® (Cedars-Sinai, Los Angeles, CA, USA) could simplify the process.

All imaging modalities, including the 18F-FDG PET imaging and further QPS/QGS® software evaluation, bear aspects of limitation and value. The QPS/QGS® software extrapolates the ventricle's contour at different levels in all axis (illustrated Figure 1C) based on tracer uptake and a normative database. However, 18F-FDG uptake is diminished in the infarct area. Thus, great infarcts (>30%) could lead to a systematic bias in the QPS/QGS® software evaluation when only based on PET without imaging the ventricle contour by MR. As seen in Figure 1C and Supplementary Figures 2A,B, we could detect no motion in the area of diminished 18F-FDG uptake arguing for a proper software evaluation based on PET. Although the software being developed for human- and modified for rodent studies, we cannot exclude perturbing factors in cardiac geometry, pixel scaling, and partial-volume effects related to smaller animal hearts. However, using the symbiosis of PET and MR in future small-animal studies could present great benefit.

Previous publications show that the infarcted area (day 7) detected by PET imaging is slightly bigger than in histology (30, 31). Our assessment at day 3 and day 30 after MI is in line with these observations. Similar to the publication of Stegger et al. and Greco et al. this could be due to different physiological conditions of *in vivo* PET compared to *ex vivo* histology and the lack of ECG gating in the assessment of infarct size (30), and therefore the missing discrimination of diastole and systole could further have impact on the assessment of infarct size. The harvested hearts were not stopped in diastole (e.g., by injection of KCL) which could interfere with our assessment. Further, this observation could be confounded by invading immune cells and sterile cardiac inflammation that cannot be differentiated by 18F-FDG (32, 33). We could already detect a strong correlation at day 3 post-operation, with an even better correlation at day 30 post-infarction. Further, Bland-Altman's calculation bias reduces from day 3 (Bias: 10.33) compared to day 30 (Bias: 5.33). Advanced histological differentiation of the infarcted area at day 30 could explain this result. Another important consideration is the reduced myocardial FDG uptake at day 30, and therefore the decrease in the spill-over effect.

The Bland Altman plot is indicating a systematic bias in comparing the two infarct assessments. Our data also indicate an overestimation of infarct area by PET imaging at higher values and underestimation at marginal infarcts. Possible differences in the anatomy of coronary arteries were not evaluated in this project. Collectively, there is a valid positive correlation between PET infarct and histology infarct size.

The metabolic volume is already a valued prognostic tool in cancer diagnostics and therapy (34, 35). The previous publication showed that the LVMV is an accurate surrogate marker for heart mass during pressure-overload left ventricular hypertrophy (12).

Previous work demonstrated a cardiac growth dependency in mice from early to mid-adulthood (24). The LVMV, estimating the cardiac mass (12) in mice, rises from 10 to 18 weeks and the ESV and EF changes over time. These observations need to be considered when monitoring the course of LVMV after myocardial infarction, thus indicating ongoing adolescent growths and compensatory cardiac remodeling.

Our infarct experiments show that the LVMV is dropping early at day 3 post-operation due to the loss of viable tissue and increases during the following month, probably by the compensatory cardiac remodeling and hypertrophic response (12). After MI, left ventricular hypertrophy is a compensatory mechanism after a cardiac injury to sustain the ventricle's stroke volume. The ongoing compensatory mechanism can, nevertheless, result in heart failure (36).

Cardiac %ID/g is another nuclear surrogate parameter in the setting of MI (20). Our data show a significant increase at day 3 post-infarct that diminishes over time. This could demonstrate a rise in metabolic activity in the injured heart in the short-term. The increased demand for 18F-FDG could be mediated by several mechanisms, e.g., inflammatory processes, invading immune cells, and increased workload (32, 37, 38). This novel surrogate marker warrants further investigations to provide insight into the uptake of 18F-FDG after myocardial infarction and the role of invading immune cells that could theoretically increase the cardiac 18F-FDG uptake. Collectively, we assume that the most relevant metabolic alterations occur between day 1 and day 14. Assessment of these specific alterations in short-interval PET evaluation could provide more insight in future investigations.

Echocardiography provides another widely used method of evaluation murine cardiac function (31). Nevertheless, echocardiography only provides a 2D imaging, and could show investigator dependent bias. Infarct size determination and evaluation of metabolic changes are not possible. Furthermore, PET imaging and MRI provide an automated process for assessing the cardiac function.

An alternative for *in vivo* small-animal infarct assessment is provided by three-dimensional late gadolinium enhancement MRI (39). For instance, Bohl et al. implemented a high-resolution method to quantify infarct size in mice. They could show an excellent correlation and agreement in Bland-Altman analysis compared to histology (40). Using MR also enables to assess of the ventricular function (EDV, ESV, LVEF) based on the direct measurement of the ventricle wall, which could overcome the limitation of 18F-FDG PET estimation in great murine myocardial infarcts (41, 42).

Besides, we estimated clinically relevant cardiac left ventricular parameters after MI at different stages. Evaluation of left ventricular function parameters by PET is comparable to MRI (21, 43, 44). Like previous publications, PET evaluation could detect increased EDV and decreased EF after MI (23, 43). Remarkably, the SV remains stable in the acute and the late stage of our experimental setting. Mathematically, the EF is defined by SV divided by EDV. Thus, the reduced contraction and emptying of the left ventricle but increased EDV leads to stable SV. The evaluation of the cardiac function in mice of different age (10 and 18 weeks old) could be influenced by adaption and repetition of isoflurane narcosis and as well due to the growth process from early- to mid-adulthood.

Yet, there are several methodical limitations in using 18F-FDG for small-animal cardiac PET imaging. Of note, the isoflurane anesthesia has been shown to significantly enhance myocardial FDG uptake in mice (45). The cardiac metabolism could be influenced by anabolic and catabolic hormones such as insulin, insulin growth-factor or glucagon, that were not evaluated in this study.

Another limitation of this work is the model of permanent LAD ligation itself. Today percutaneous coronary intervention (PCI) to re-open vessels differs pathophysiological from the model used in this research project. After revascularization, novel challenges such as the reperfusion injury in the heart tissue occur (46). Further, it should be considered that the mice used for the MI are healthy and still in their early adulthood. Compared to patients in the clinic, missing comorbidities such as hypertension, diabetes, and atherosclerosis could impact cardiac injury and remodeling.

Future murine infarct PET studies could tremendously benefit from biomarkers that focus on cardiac hypoxia [reviewed in (47)] and novel promising tracer such as 18F-fluoromisonidazole (FMISO) (48) or calculation of myocardial oxidative metabolism by 11C-acetate PET scans (49, 50).

Collectively, we showed that the nuclear medicine markers of LVMV and cardiac %ID/g could act as possible parameters to assess the acute myocardial injury. The LVMV may especially display the opportunity to detect the left ventricle's mass by quantification of the metabolic 18F-FDG uptake. Therefore, these new parameters warrant further investigation in, e.g., a cardiac ischemia-reperfusion injury model.

Our results support the notion that 18F-FDG PET imaging is useful and valid for infarct area detection and evaluation, without the need of sacrificing mice at different stages of the experimental setting.

## Data Availability Statement

The original contributions presented in the study are included in the article/Supplementary Material, further inquiries can be directed to the corresponding author/s.

## Ethics Statement

The animal study was reviewed and approved by Government's animal ethics committee (Gz. 55.2-1-54-2532-45-13).

## Author Contributions

MF and AT: conceptualization. AT and LP: methodology. MZ, LP, and LW: validation. MF: investigation. KS: data curation. MF: writing—original draft preparation. AT: writing—review and editing. TW and KS: visualization. PB and SL: supervision. CS and AT: project administration. MF, CS, and SM: funding acquisition. All authors have read and agreed to the published version of the manuscript.

## Conflict of Interest

The authors declare that the research was conducted in the absence of any commercial or financial relationships that could be construed as a potential conflict of interest.
