# Supplementary material for: Cardiac 18F-FDG Positron Emission Tomography: An Accurate Tool to Monitor In vivo Metabolic and Functional Alterations in Murine Myocardial Infarction
Source: Front Cardiovasc Med. 2021 May 25;8:656742. doi: 10.3389/fcvm.2021.656742 (PMC8185215; doi:10.3389/fcvm.2021.656742)
Supplement: Supplementary file 1 [file Data_Sheet_1.docx]

Supplementary Material

# Supplementary and Tables

**Suppl. Table S1: Overview of experimental infarct groups of different ages**

|  | **Group 1** | **Group 2** |
| --- | --- | --- |
| **Mice age** | **10 weeks old** | **18 weeks old** |
| **PET Scan at Baseline** | Yes | Yes |
| **1. PET Scan: 3d pOP** | Yes/subgroup | Yes |
| **2. PET Scan: 14d pOP** | Yes/ subgroup | No |
| **3. PET Scan: 30d pOP** | Yes/ subgroup | No |
| **Histology 3d pOP** | Yes/subgroup | Yes |
| **Histology 30d pOP** | Yes/subgroup | No |

**Suppl. Table S2: PET measurements in 18 weeks old mice after myocardial infarction**

|  | **PET Infarct/ defect (%)** | **LVMV (mm^3^)** | **%ID/g** | **EDV (µl)** | **ESV (µl)** | **SV (µl)** | **EF (%)** |
| --- | --- | --- | --- | --- | --- | --- | --- |
| Baseline | 2.1 ± 2.1 | 200.9 ± 14.7 | 31.5 ± 5.9 | 44.9 ± 5.7 | 8.2 ± 3.2 | 36.8 ± 3.1 | 82.1 ± 5.3 |
| 3d pOP | 39.3 ± 6.5 | 164.7 ± 12.7 | 46.1 ± 5.6 | 54.4 ± 6.7 | 30.3 ± 5.9 | 23.9 ± 2.5 | 44.1 ± 6.0 |

Values indicate Mean ± SD. Defect, LVMV left ventricular metabolic volume, %ID/g percentage of the injected dose per gram, EDV end-diastolic volume, ESV end-systolic volume, SV stroke volume, EF ejection fraction.

## Supplementary Figure

**Suppl. Figure S1.**

**(A)** Schematic study design of the induction of myocardial infarct (MI), PET imaging and histology.

**(B)** Histogram of the infarct area (in %) in histology and PET imaging according to day 3 and day 30 post-MI in experimental group 1 and 2.

**(C)** Comparison of cardiac PET parameters at baseline (in green), and 3 days post-infarct (in red) in 18 weeks old male mice. All groups: n = 7-8. Data represent mean ± SD. * p < 0.05, ** p < 0.01, *** p < 0.001.

**Suppl. Figure S2.**

**(A)** Segmental defect scoring of three representative infarct hearts from QPS software. Color scale from QPS in Cool and percentage range. Bull’s Eye Plot according to the AHA-conform nomenclature depict segmental defects in myocardial segments 17, 16, 13, and 7.

**(B)** Correlating segmental motions severity scoring in standard deviation (SD) from QGS software. Bull’s eye plot according to the AHA- conform nomenclature.
